# Childhood Reports of Food Neglect and Impulse Control Problems and Violence in Adulthood

**DOI:** 10.3390/ijerph13040389

**Published:** 2016-03-30

**Authors:** Michael G. Vaughn, Christopher P. Salas-Wright, Sandra Naeger, Jin Huang, Alex R. Piquero

**Affiliations:** 1 School of Social Work, College for Public Health and Social Justice, Saint Louis University, St. Louis, MO 63103, USA; naegersr@slu.edu (S.N.); jhuang5@slu.edu (J.H.); 2 School of Social Work, University of Texas at Austin, Austin, TX 78712, USA; salaswright@utexas.edu; 3 Program in Criminology, School of Economic, Political and Policy Sciences, University of Texas at Dallas, Dallas, TX 75080, USA; apiquero@utdallas.edu

**Keywords:** interpersonal violence, food neglect, self-control, reactive aggression, violence

## Abstract

Food insecurity and hunger during childhood are associated with an array of developmental problems in multiple domains, including impulse control problems and violence. Unfortunately, extant research is based primarily on small convenience samples and an epidemiological assessment of the hunger-violence link is lacking. The current study employed data from Wave 1 (2001–2002) and Wave 2 (2004–2005) of the National Epidemiologic Survey on Alcohol and Related Conditions (NESARC). The NESARC is a nationally representative sample of non-institutionalized U.S. residents aged 18 years and older. Participants who experienced frequent hunger during childhood had significantly greater impulsivity, worse self-control, and greater involvement in several forms of interpersonal violence. These effects were stronger among whites, Hispanics, and males. The findings support general theoretical models implicating impulse control problems as a key correlate of crime and violence and add another facet to the importance of ameliorating food neglect in the United States.

## 1. Introduction

The United States Department of Agriculture estimates that more than 15 million children in the United States reside in households facing challenges related to food security. Food security refers to the consistent access to sufficient and adequate nutrition and the lack thereof—experiencing frequent hunger—has manifold implications for healthy child and adult development [1,2,3,4,5]. Indeed, it is well-documented that inadequate nutrition stemming from food insecurity is associated with outcomes such as poor health, asthma and other chronic health conditions, and frequent hospitalizations as well as challenges with respect to neuro-cognitive, academic, psycho-emotional, and social skills development [6,7,8,9,10,11,12,13,14]. Fundamentally, a robust body of research suggests that hunger and inconsistent access to nutritious food whether by purposeful neglect or not have important implications with respect to healthy growth and human development.

Inadequate childhood nutrition can be the result of material deprivation that is part and parcel of family-of-origin poverty, the result of parental neglect whereby the basic parent is unwilling or unable to provide basic life necessities for the child, or a combination of both. A large literature has shown that parenting deficits contribute to impulse-control deficits in children for a variety of reasons [6,15,16,17,18]. Moreover, a recent study reported evidence that sleep deprivation was significantly associated with childhood low self-control and indirectly related to delinquency *via* these same impulse control mechanisms [19].

While much research has been conducted on the links between food insecurity and the aforementioned health and developmental outcomes, fewer studies have examined the links between childhood food neglect and interpersonal violence. We define food neglect as an adverse childhood experience that occurs when a parent, caregiver, or other adult living in a home makes a child go hungry or does not prepare regular meals. Childhood behavioral problems—often a precursor for later antisocial behavior and violence—have been linked with nutritional inadequacies [20]. More specifically, poor nutrition early in life has been linked to deficits in executive functioning such as attention deficit hyperactivity disorder (ADHD) and attentional problems [21,22] in childhood and adolescence that are more likely to occur among males. For instance, Lukowski and colleagues [23] found that infant iron deficiency was predictive of poor results during early adulthood with respect to long-term planning and behavioral inhibition. Studies of inadequate nutrition and antisocial behavior have found associations with aggressive and/or externalizing behaviors [5,24,25,26,27,28]. Finally, in a randomized controlled trial of vitamin and mineral supplementation in 6–12 year olds who engaged in disciplinary infractions (e.g., fighting, being disrespectful, vandalism, obscenities), Schoenthaler and Bier [29] found that the supplemented treatment group had a 47% lower rate on average of antisocial behavior than controls. Two other studies have also reported an association between exposure to violence and food insecurity [30,31]. Nutrition clearly matters in relation to impulse control.

Healthy brain development is fueled not only by key micronutrients [3] but also adequate caloric intake to provide energy. Areas of the brain involved with behavioral inhibition (*i.e.*, frontal areas) may be particularly sensitive to the effects of inadequate nutrition [21,22,26]. The brain consumes a substantial proportion of the body’s calories (approximately 20%) and energy from glucose is critical to its functioning [32]. One theoretical mechanism by which inadequate nutrition exerts its effects on violence is by diminishing impulse-control. Several theoretical perspectives cite impulse control deficits as a core etiology basis of antisocial behavior and criminal offending [33,34,35,36,37,38,39]. While these theories often acknowledge early-life abuse and deprivation in the backgrounds of individuals with impulse control deficits, they often do not explicitly acknowledge food insecurity, hunger, and nutritional deprivation.

Lack of food has been found to be related to poor glucose control [40]. Recent studies of the self-control strength model build on this basic finding and have shown that the ability to maintain impulse-control is dependent on energy derived from adequate levels of glucose [41,42,43] and that low glucose levels are in turn associated with increased aggression [44]. Moreover, as DeWall and colleagues [44] reported in a correlational cross national study, nations that have a higher prevalence of individuals with interrupted glucose metabolism stemming from glucose-6-phosphate dehydrogenase deficiency are involved in a greater degree of non-war violence. An alternative interpretation should be acknowledged and this involves children who are exposed to stress or deficiencies in the rearing environment (such as nutritious food) as they tend to also receive less cognitive and emotional stimulation, which may result in reduced impulse regulation [45].

### The Current Study

Although several studies point to a relationship between food neglect, inadequate nutrition and poor outcomes, relatively few investigations have examined the relationship between food neglect during childhood and later impulse control deficits and violence in adulthood. Meanwhile, there have been a number of studies that have demonstrated the importance of energy in the form of glucose in maintaining self-control strength in relation to aggression [2,44,46], though recent and much publicized replication efforts have been disappointing. Further, as mentioned previously, the food neglect-aggression link can also be due to the impact of the deficient rearing environment [45]. The major weaknesses found in previous work are that most of these studies have employed convenience samples or geographically circumscribed data sources that may lack adequate generalizability. If going hungry leads to restricted caloric intake then there should be less energy available to maintain impulse control. This may be especially problematic for males given the higher probability of externalizing behavior compared to females [47]. The present study builds on and extends prior research on food neglect, violence, and impulse-control by examining interpersonal violence and impulse-control deficits among males and females who reported experiencing frequent hunger in data derived from a population-based sample. An additional advantage of employing a population-based sample is the available power and representativeness allows analyses to stratify by both gender and racial and ethnicity permitting meaningful comparisons. We hypothesize that childhood experiences of food neglect in the form of experiencing frequent hunger will be associated with not only poor impulse control, but also a higher likelihood of violence even after controlling for numerous sociodemographic and behavioral confounds. In addition, based on a higher prevalence of externalizing found among males in general we hypothesize that effects will be stronger for males than females. Our analyses comparing racial and ethnic differences are exploratory and descriptive and thus no hypotheses are proffered.

## 2. Materials and Methods

### 2.1. Participants

Study findings are based on data from Wave 1 (2001–2002) and Wave 2 (2004–2005) of the National Epidemiologic Survey of Alcohol and Related Conditions (NESARC). The NESARC is a nationally representative sample of non-institutionalized U.S. residents aged 18 years and older. The survey gathered data from individuals living in households and group settings such as shelters, college dormitories, and group homes in all 50 states and the District of Columbia. The NESARC utilized a multistage cluster sampling design, oversampling young adults, Hispanics, and African-Americans to ensure appropriate representation of racial and ethnic subgroups and obtain reliable statistical estimation in these subpopulations. The NESARC measures are not entirely consistent across the two waves of data collection. As such, consistent with previous research using the NESARC [48,49], data from Waves 1 and 2 were combined in order to assess the associations between a broader range of constructs. The response rate for Wave 1 data was 81% and for Wave 2 was 87% with a cumulative response rate of 70% for both waves and adjustments made for nonresponse [48].

Multistage cluster sampling design is a commonly used design when attempting to provide nationally representative estimates. In the NESARC, a total of 709 primary sampling units (PSUs) provided by the Census Supplementary Survey were selected (Stage One). Within the sample PSUs, households were systematically selected (Stage Two). An individual age 18 or older was randomly selected from each household. Data were weighted at the individual and household levels to adjust for oversampling and non-response on demographic variables (*i.e.*, age, race/ethnicity, sex, region, and place of residence). The sample was slightly more female (52.1%) than male (47.9%) and the mean age of the sample is 49.1 years (Standard Deviation = 17.3) with ages at Wave 2 ranging from 20 to 90. A majority of the sample reported having completed some college or more (58.5%) and being currently married (63.8%), and roughly one in three respondents (29.3%) reported residing in household earning $70,000 or more. A more comprehensive portrait of the sociodemographic characteristics of study participants is available elsewhere [50,51]. Data were also adjusted to be representative (based on region, age, race, and ethnicity) of the U.S. adult population as assessed during the 2000 Census. NESARC is an extant data file that is considered restricted access. The study team has met all institutional requirements with respect to human subjects. All subjects gave their informed consent for inclusion before they participated in the study. The study was conducted in accordance with the Declaration of Helsinki, and the protocol was approved by the Ethics Committee of the United States National Institutes of Health. Given that the data is pre-collected and stripped of all identifiers by NIAAA, the institutional review board (IRB) at the corresponding universities deemed the use of the NESARC data for research purposes to be exempt from IRB review. 

### 2.2. Diagnostic Assessment

Data were collected through face-to-face structured psychiatric interviews conducted by U.S. Census workers trained by the National Institute on Alcohol Abuse and Alcoholism and U.S. Census Bureau. Interviewers administered the Alcohol Use Disorder and Associated Disabilities Interview Schedule—DSM-IV version (AUDADIS-IV), which provides diagnoses for mood, anxiety, personality, and substance use disorders [52]. The AUDADIS IV demonstrates good-to-excellent reliability in assessing alcohol and drug use in the general population [53,54]. Specific disorders included major depression, dysthymia, and bipolar disorder, social phobia, generalized anxiety disorder, panic disorder, specific phobia, and psychotic disorder. In addition to the assessment of psychiatric disorders a host of sociodemographic and background characteristics were also collected in these interviews. These included age, gender, race and ethnicity, income, education, marital status, and region of the country. The response rate for Wave 1 data was 81% and for Wave 2 was 87% with a cumulative response rate of 70% for both waves.

### 2.3. Measures

#### 2.3.1. Food Neglect/Frequent Hunger 

Respondents were asked: “How often did a parent or other adult living in your home make you go hungry or not prepare you regular meals?” This variable was dichotomized to facilitate a clear distinction between the presence and absence of consistent or frequent exposure to childhood hunger. Individuals reporting having experienced hunger “fairly often” or “often” were coded as 1 and all other individuals (*i.e.*, those reporting having “never”, “almost never”, or “sometimes” experienced hunger) were coded as 0. An alternative coding strategy would be to categorize individuals reporting “sometimes” experiencing hunger with those reporting “fairly often” or “often” experiencing hunger; however, we elected to prioritize recurrent experiences of hunger by drawing a sharp contrast between individuals who reported experiencing frequent hunger *versus* all other individuals. While it is optimal to measure hunger using multi-item measures, epidemiological studies have made use of similar single-item measures [12,55]. Retrospective accounts of frequent hunger were measured during the Wave 2 assessment only.

#### 2.3.2. Interpersonal Violence

Four dichotomous (0 = no, 1 = yes) measures from the antisocial personality disorder module of the Alcohol Use Disorder and Associated Disabilities Interview Schedule—DSM-IV version (AUDADIS-IV) were used to examine violent behavior. While the AUDADIS-IV includes items measuring an array of antisocial behaviors, we selected only items that explicitly measured intentional acts of interpersonal violence. Data from Waves 1 and 2 were combined to measure respondent self-report of having exhibited any of the behaviors in their lifetime. Items include: “In your entire life, did you ever physically hurt another person in any way on purpose?”, “In your entire life, did you ever hit someone so hard that you injured them or they had to see a doctor?”, “In your entire life, did you ever use a weapon like a stick, knife, or gun in a fight?” and “In your entire life, did you ever physically hurt another person in any way on purpose?” We also created a variable for “any violence” in which individuals responding affirmatively to one or more violence measures were coded as 1 and those reporting no involvement in violence were coded as 0.

#### 2.3.3. Impulse-Control Deficits 

Four dichotomous (0 = no, 1 = yes) variables related to impulse-control deficits as measured in the borderline personality disorder module of the AUDADIS-IV were also utilized. Respondents were asked to consider impulse-control in terms of long-term patterns of behavior (*i.e.*, “Most of the time throughout your life, regardless of the situation or whom you were with”). Items include: “Have you often done things impulsively?”, “Have even little things made you angry or have you had difficulty controlling your anger?”, “Have you often had temper outbursts or gotten so angry that you lose control?”, and “Have you hit people or thrown things when you got angry?” We also created a variable for “any self-control deficits” in which individuals responding affirmatively to one or more of the impulse-control deficit measures were coded as one and all others coded as 0. Items related to impulse-control deficits were measured during the Wave 2 assessment only.

#### 2.3.4. Mental and Substance Use Disorders 

In all multivariate analyses, we controlled for lifetime DSM-IV clinical, personality, and substance use disorders on the basis of the Alcohol Use Disorder and Associated Disabilities Interview Schedule—DSM-IV version (AUDADIS-IV), which has been shown to have good-to-excellent reliability in the diagnosis of mental disorders in the general population [48,53].

#### 2.3.5. Sociodemographic Factors 

The following sociodemographic variables were included as controls: age (18 to 34 years, 35 to 49 years, 50 to 64 years, 65 years and older), gender (male, female), race/ethnicity (non-Hispanic white, African-American, Hispanic, other (e.g., Asian/Pacific Islander, Native American/Alaska Native), household income (less than $20,000; $20,000–$34,999; $35,000–$69,999; greater than $70,000 per year), education level (less than high school, high school graduate only, attended some college, completed associate’s, bachelor’s, or technical degree), region of the United States (Western, Northeastern, Midwestern, or Southern United States), and urbanicity (rural, urban). Consistent with prior research using the NESARC [44], we coded age and family income as categorical variables. All sociodemographic variables were taken from the Wave 2 data in order to conduct analyses with the most up-to-date information. We also controlled for self-reported parental history of antisocial behaviors (*i.e.*, “In your judgment, did your blood or natural father/mother have behavior problems (cruelty to animals, impulsive or destructive behavior, violence and crime, *etc.*) at any time in his/her life?” and drug abuse (*i.e.*, “In your judgment, has your blood or natural mother/father had problems with drugs at any time in his/her life?”), both of which were measured during the Wave 1 assessment only.

### 2.4. Analysis

**Figure 1:**
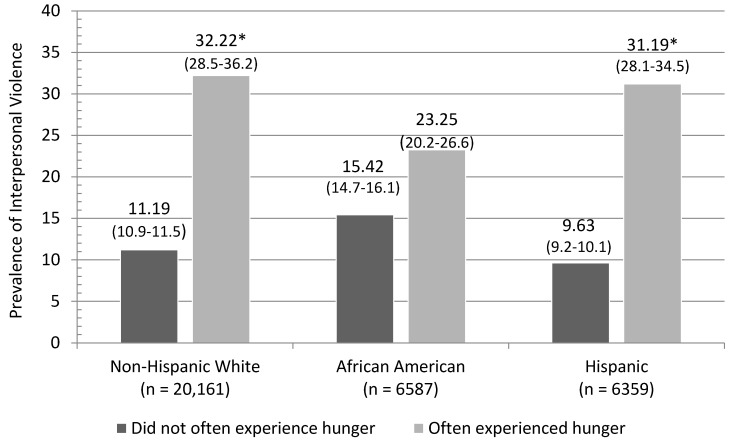
Survey adjusted prevalence estimates of interpersonal violence among individuals who often experienced hunger by race/ethnicity. Differences significant (*p* < 0.05) while controlling for sociodemographic, parental, and psychiatric confounds are indicated by the use of an asterisk (*) next to the higher prevalence estimate.

Statistical analyses were carried out in multiple steps. First, logistic regression analyses were conducted that compared individuals reporting frequent exposure to hunger during childhood with individuals in the general population with respect to sociodemographic factors (Table 1) and violent antisocial behavior (Table 2). Next, we examined the relationship between frequent hunger and violence across gender (Table 3) and among non-Hispanic white, African-American, and Hispanic respondents (Figure 1) using stratified logistic regression analysis. In keeping with previous research, we examined the overlapping/no overlapping confidence intervals in order to test the differences in the magnitude effects across gender and race/ethnicity [56] With categorical data, this approach is preferable to more traditional (*i.e.*, multiplicative) tests of moderation due to the potential for unequal residual dispersion between groups [57]. Finally, we used logistic regression analysis to examine the relationship between frequent hunger and variables related to long-term patterns of impulse-control deficits (Table 4). Adjusted odds ratios (AORs) were considered to be statistically significant if the associated confidence intervals did not cross the 1.0 threshold. For all statistical analyses, weighted prevalence estimates and standard errors were computed using Stata 13.1 SE software (StataCorp LP, College Station, TX, USA) [58]. More information on NESARC waves 1 and 2 data can be found here http://www.psc.isr.umich.edu/dis/data/catalog/detail/1179 [59].

## 3. Results

### 3.1. Characteristics of Individuals Reporting Frequent Exposure to Hunger

Table 1 compares individuals reporting frequent exposure to hunger during childhood with individuals in the general population with respect to sociodemographic factors. Individuals reporting frequent hunger were significantly more likely to be under the age of 65, to reside in a household earning less than $70,000 per year, and to have earned less than a college degree. Individuals experiencing frequent hunger were also significantly less likely to be female (AOR = 0.64, 95% CI = 0.58–0.72). Compared to non-Hispanic whites, individuals reporting frequent hunger were significantly less likely to be African-American (AOR = 0.72, 95% CI = 0.62–0.84) and significantly more likely to be Hispanic (AOR = 1.31, 95% CI = 1.12–1.54) or of “other” race/ethnicity (AOR = 1.94, 95% CI = 1.68–2.24).

### 3.2. Is Frequent Hunger During Childhood Associated with Interpersonal Violence?

Table 2 compares individuals reporting frequent exposure to hunger during childhood with individuals in the general population with respect to aggressive behavior. Controlling for age, gender, race/ethnicity, household income, education level, region of the United States, urbanicity, antisocial parental history, and lifetime clinical, personality disorders, and alcohol and drug use disorders, individuals reporting frequent hunger during childhood were significantly more likely to report a lifetime history of interpersonal violence (AOR = 2.08, 95% CI = 1.81–2.38). Significant associations were also found for all individual manifestations of interpersonal violence examined, including fight starting (AOR = 2.18, 95% CI = 1.77–2.68), injuring another person so that they required medical attention (AOR = 2.21, 95% CI = 1.85–2.64), use of a weapon in a fight (AOR = 2.34, 95% CI = 1.90–2.88), and physically hurting another person on purpose (AOR = 2.06, 95% CI = 1.79–2.38) (see Appendix).

### 3.3. Does the Relationship Vary Across Gender and Race/Ethnicity?

Table 3 examines the link between frequent exposure to hunger during childhood and aggressive behavior among men and women. Across gender, the link between frequent childhood hunger and violence was significant in general and for each of the specific measures of interpersonal violence. Notably, as evidenced by the non-overlapping 95% confidence intervals, the magnitude of the relationship between frequent childhood hunger and involvement in any violent behavior was greater among men (AOR = 2.73, 95% CI = 2.15–3.45) than among women (AOR = 1.63, 95% CI = 1.36–1.96). Significant gender differences were also observed for fight starting (Male: AOR = 3.07, 95% CI = 2.16–4.34; Female: AOR = 1.41, 95% CI = 1.07–1.5), but not for the other specific manifestations of interpersonal violence.

Figure 1 presents the prevalence estimates for involvement in aggressive behavior among individuals reporting frequent exposure to hunger during childhood, by race/ethnicity. The raw prevalence estimates for interpersonal violence are higher among those reporting frequently experiencing childhood hunger as compared to those who did not often experience hunger early on in life. However, controlling for sociodemographic, parental, and mental health confounds, we found a significant association between experiences of hunger and interpersonal violence among non-Hispanic whites (AOR = 2.29, 95% CI = 1.86–2.82) and Hispanics (AOR = 2.57, 95% CI = 2.21–3.00), but no significant association was identified for African-Americans (AOR = 0.75, 95% CI = 0.53–1.06).

### 3.4. Is There a Link Between Frequent Hunger and Impulse-Control Deficits?

Table 4 examines the relationship between frequent hunger and variables related to long-term patterns of impulse-control deficits. Controlling for the same array of sociodemographic, parental, and psychiatric confounds, individuals reporting frequent hunger during childhood were significantly more likely to report challenges related to impulse-control (AOR = 1.97, 95% CI = 1.70–2.29). Specifically, those experiencing frequent childhood hunger were significantly more likely to report having often behave impulsively (AOR = 1.70, 95% CI = 1.45–2.00), difficulty controlling anger (AOR = 1.19, 95% CI = 1.04–1.36), frequent temper outbursts (AOR = 1.81, 95% CI = 1.61–2.03), and violent behavior when angry (AOR = 1.66, 95% CI = 1.39–1.98). We also conducted supplementary analyses to examine potential gender and racial/ethnic differences in the relationship between hunger and impulse-control deficits. The relationship was significant among both male (AOR = 2.24, 95% CI = 1.72–2.93) and female (AOR = 1.76, 95% CI = 1.56–2.00) respondents with overlapping confidence intervals pointing to no significant gender difference. In terms of race/ethnicity, the link between hunger and impulse-control deficits was significant for non-Hispanic white (AOR = 2.11, 95% CI = 1.69–2.63) and Hispanic (AOR = 1.70, 95% CI = 1.48–1.92) but not African-American respondents.

We also conducted an exploratory analysis using structural equation modeling in which we examined impulse-control deficits as a mediating factor between frequent hunger and interpersonal violence (χ^2^ = 2395.77 (DF = 121)), RMSEA = 0.024 (90% CI = 0.023–0.025), CFI = 0.951, TLI = 0.934). Controlling for sociodemographic, parental, and psychiatric factors, the results of our modeling suggest that frequent hunger is associated with impulse-control deficits (Beta = 0.04, *p* < 0.001) which, in turn, is associated with interpersonal violence (Beta = 0.25, *p* < 0.001). We also identified a direct path from frequent hunger to interpersonal violence (Beta = 0.06, *p* < 0.001). We also ran the model without the variable that referenced “hitting people” and found very similar results (Beta = 0.21, *p* < 0.001). While suggestive, the results of the these exploratory analyses should be interpreted judiciously as the cross-sectional nature of the NESARC data—and the measurement of key variables used in this study (e.g., hunger, impulse-control, and interpersonal violence)—is such that mediation is difficult to meaningfully examine.

## 4. Discussion

The importance of exercise and nutrition is a virtual truism in the physical, health, and social sciences. Yet, much of this line of research has been concentrated in disciplinary silos. In this study, we were concerned with the linkages between childhood food neglect, impulse control, and interpersonal violence, thereby helping to bridge the gaps between research in the health sciences, psychology, and criminology.

Prior research has suggested that impulse control is partly dependent on adequate levels of glucose [41,42,43,44] and therefore experiencing frequent hunger makes it more difficult to maintain impulse-control. In another conceptual model, children who are exposed inadequate nurturing environments that may include food neglect incur less cognitive and emotional stimulation resulting in impaired impulse regulation [45]. As Miller and colleagues’ state, “The model postulates that childhood stress also shapes the functioning of the neural circuitry that underlies self-regulation of appetitive behaviors. This shaping process gives rise to a phenotype that highly discounts the future, behaves in an impulsive fashion, and readily seeks out appetitive stimuli. As a consequence, the individual has a propensity for engaging in health-compromising behaviors, like smoking cigarettes, eating high-fat foods, avoiding physical activity, and drinking excess alcohol” ([45], p. 980). Consistent with these lines of theorizing, we found significant associations between frequent childhood hunger and measures of impulse-control deficits. We also found that frequent experiences of hunger during childhood are associated with the increased likelihood of involvement in an array of violent behaviors, especially for males. Indeed the prevalence of lifetime involvement in interpersonal violence among those reporting having frequently gone hungry (37%) was nearly twice that of those who reported little-to-no experience of childhood hunger (15%). A similar pattern was observed not only for interpersonal violence in general, but for specific manifestations of violence such as fight starting, injuring someone in a violent altercation, the use of a weapon in a fight, and the purposeful injury of another person. Notably, the link between childhood hunger and each of these manifestations of interpersonal violence was found to be significant while controlling for a host of sociodemographic, parental, and lifetime psychiatric and substance use disorder confounds. Findings suggest that the consistent experience of hunger during childhood may place individuals at risk for impulsive behavior as well as temper outbursts and difficulty refraining from violent behavior when angry, especially among those with difficult temperaments [34,60,61,62,63,64]. This finding is also consistent with research showing that hypoglycemia and reduced L-tryptophan concentration is associated with impulsive, reactive aggression [29,65,66,67,68,69].

Beyond examining the main relationship between frequent hunger and interpersonal violence among all study participants, we also examined the degree to which this relationship was moderated by gender. Findings indicated that childhood hunger was significantly associated with increased likelihood of interpersonal violence among both men and women; however, consistent with our hypothesis the hunger-violence link was more pronounced among men than among women. This pattern of effects was particularly evident in terms of frequent fight starting which, notably, is the measure of violence that most clearly captures violent behavior in which the individual is the aggressor and is not potentially responding in self-defense. Our results showed a clear distinction in starting fights as evidenced by the non-overlapping confidence intervals among males and females. Research has found that males are more likely to engage in physical altercations that escalate into violence [47]. Cross, Copping, and Campbell [70] in their meta-analysis of 277 studies found that women had better effortful control than men, which may serve to buffer them for escalating aggression. Importantly one more possible health benefit for ameliorating food insecurity is the potential reduction in male violence.

We also found evidence of a marked pattern of differences with respect to race/ethnicity that are less obvious to interpret compared to male-female differences. Namely, childhood hunger was significantly associated with the increased likelihood of interpersonal violence among non-Hispanic white and Hispanic respondents, but no significant relationship was found among African-Americans. One possibility is that this could be an artifact of survey sampling or that African-Americans are more likely to report having access to calories and therefore not go hungry even if that food is high in fat and lacks nutrient density. Research of racial and ethnic differences has found that African-Americans are more likely to be obese compared to Whites and Hispanics [5,71]. There may also be socioeconomic status and race/ethnicity confounding in that lower income persons in the sample uniformly were more likely to report going hungry. Another corollary perspective is offered by Mischel [72] who has found that under harsher social conditions one would expect actions based on satisfying immediate survival needs thus resulting in delay of discounting and reduced impulse-control.

Over the past decade there has been an increased recognition that violence can be usefully conceptualized as a public health issue. The consequences of hunger, whether by neglect or lack of resources to obtain food, are widespread but one major implication of the present paper is that ameliorating hunger can potentially reduce violence. As such, within the public health approach to violence hunger is a candidate risk factor. Although poor impulse-control is a robust correlate of crime, violence, and many other adverse outcomes [73], research suggests that energy from adequate nutrition can help to maintain impulse-control. Thus, strategies that alleviate hunger and increase food security in turn can help to deflect one of the proximal mechanisms for violence, namely poor impulse-control. And while most evidence-based programs focusing on improving impulse-control are focused primarily on parental (and to a lesser extent teacher) socialization efforts [74], it seems that educating parents about more holistic socialization to include exercise and especially diet and nutrition is relevant. Importantly, from a political perspective hunger reduction strategies are likely to be supported from both the right and the left.

Taken into consideration with other recent studies regarding food scarcity, our results suggest that social services and public health agencies should undertake efforts to minimize the adverse effects of food scarcity and its by-product of poor nutrition. Our findings show that persons who do not have the necessary quantity and quality of food face adverse effects that may reverberate adversely over the life course. Our findings also suggest an expanding research agenda going forward. One study in particular would examine what has been commonly referred to as food deserts—particularly in disadvantaged communities in large urban cities [75]. The interaction between food deserts, poor glucose control, and penultimately deficits in impulse-control and compromised educational opportunities suggests that certain persons in certain locations may be at heightened risk for adverse consequences throughout the life course. Identification of these risk factors and empirically assessing their effects is not only relevant for theoretical and empirical work in this area, but can also help identify additional policy-relevant proscriptions for certain communities.

### Study Limitations

Study findings should be interpreted in light of several important limitations. First, the NESARC is not a true longitudinal study and, as such, the temporal ordering of the measurement of frequent exposure to childhood hunger and interpersonal violence is less than optimal. The use of a prospective study design would be optimal in terms of assessing the longitudinal associations between the experiences of hunger in childhood and violent behavior. Moreover, we necessarily relied upon retrospective reports and for some study participants this stretched the recall period over several decades. Further, there was no challenge test involving fasting and the administration of glucose or other food to experimentally assess impulse control. Second, the measures of hunger and interpersonal violence are derived exclusively from the self-report data. As such, it is possible that individuals may have under or over reported to respect to the frequency of childhood hunger and the involvement in various manifestations of violent behavior. Also, we relied on less robust single item indicators and the wording of the hunger item perhaps suggested willful neglect rather than hunger due to poverty, though poverty and neglect are often associated. Additional wording problems are found in the relations between anger and impulse-control. For example, one of the impulse-control items included the wording “gotten so angry that you lose control…” introduces a tautological process in that anger is associated with violence. It should also be noted that many statistical tests were conducted in this study and, as such, there is the potential for Type I errors. Finally, the NESARC does not include variables relating to precipitating, situational, or other biological factors that may help to explain the relationship between hunger, self-control, and violence. Future research on the relationship between the nutritional influences on impulse-control and violence would gain tremendous insight into the incorporation of such factors into study designs. 

## 5. Conclusions

The present study findings point to a link between frequent childhood hunger and involvement in interpersonal violence. Indeed, controlling for an array of sociodemographic, parental, and psychiatric confounds, individuals who reported having frequently gone hungry during childhood were significantly more likely to report having started a lot of fights, injured someone in a violent altercation, use a weapon in a fight, or physically hurt another person on purpose. Notably, the links between frequent hunger and interpersonal violence were significantly more robust among men than women. Additionally, important differences were observed between racial/ethnic groups as robust effects were observed for non-Hispanic whites and Hispanics, but no significant relationship was found for African-American respondents. As hypothesized, we also found a significant link between frequent childhood hunger and challenges related to impulse-control. This suggests that, consistent with prior research and theorizing, the impact of hunger on neurocortical development and functioning may have a hand in explaining the link between hunger and interpersonal violence. Finally, results further suggest that violence can be potentially reduced by the public health strategies aimed at ameliorating hunger, which in turn, appears to be a factor involved in the development of impulse control.

## Figures and Tables

**Table 1:** Sociodemographic characteristics of individuals who often experienced hunger during childhood in the United States.

	Often Experienced Frequent Hunger? *(“How Often Did a Parent or Other Adult Living in Your Home Make You Go Hungry or Not Prepare You Regular Meals?”)*				Unadjusted		Adjusted	
Sociodemographic Factors	No (*n* = 33,991, 98.4%)		Yes (*n* = 436, 1.16%)					
	%	95% CI	%	95% CI	OR	(95% CI)	OR	(95% CI)
*Age*								
18–34 years	25.47	(25.11–25.83)	24.45	(22.24–26.80)	1.13	(0.93–1.37)	1.53	(1.25–1.88)
35–49 years	31.11	(30.79–31.44)	31.33	(29.18–33.57)	1.18	(0.99–1.42)	1.80	(1.49–2.17)
50–64 years	24.13	(23.82–24.45)	27.80	(25.67–30.05)	1.35	(1.12–1.64)	1.96	(1.61–2.38)
65 and older	19.29	(19.05–19.53)	16.41	(14.18–18.91)	1.00		1.00	
*Gender*								
Female	51.94	(51.61–52.27)	63.30	(60.73–65.79)	1.00		1.00	
Male	48.06	(47.73–48.39)	36.70	(34.21–39.27)	0.63	(0.56–0.70)	0.64	(0.58–0.72)
*Race/Ethnicity*								
Non-Hispanic White	70.67	(72.19–73.14)	65.09	(62.75–67.37)	1.00		1.00	
African American	11.29	(10.89–11.71)	9.88	(8.63–11.28)	0.98	(0.84–1.13)	0.72	(0.62–0.84)
Hispanic	4.31	(4.19–4.44)	5.21	(4.90–5.53)	1.89	(1.67–2.13)	1.31	(0.12–1.54)
Other	11.37	(11.53–11.93)	19.82	(18.13–21.63)	2.10	(1.82–2.43)	1.94	(1.68–2.24)
*Household Income*								
<$20,000	19.37	(19.10–19.65)	31.16	(28.55–33.88)	2.81	(2.49–3.18)	2.27	(2.02–2.55)
$20,000–$34,999	18.77	(18.46–19.08)	27.80	(25.19–30.58)	2.59	(2.24–3.00)	2.22	(1.95–2.54)
$35,000–$69,999	32.37	(32.07–32.67)	24.19	(21.66–26.91)	1.31	(1.13–1.52)	1.20	(1.03–1.39)
>$70,000	29.49	(29.22–29.76)	16.86	(15.47–18.33)	1.00		1.00	
*Education Level*								
Less than H.S.	13.78	(13.52–14.05)	28.82	(25.61–32.25)	3.16	(2.63–3.79)	2.32	(1.91–2.82)
H.S. Graduate	27.43	(27.06–27.81)	30.34	(27.34–33.53)	1.67	(1.45–1.93)	1.42	(1.23–1.64)
Some College	21.77	(21.47–22.07)	16.35	(14.75–18.08)	1.13	(0.97–1.33)	1.01	(0.86–1.18)
Completed AA, BA, or Technical Degree	37.01	(36.64–37.38)	24.49	(22.29–26.83)	1.00		1.00	
*Region of U.S.*								
West	17.75	(17.33–18.18)	22.44	(20.26–24.77)	1.00		1.00	
Northeast	18.53	(18.19–18.88)	16.61	(14.71–18.70)	1.18	(1.01–1.39)	1.21	(1.03–1.42)
Midwest	38.42	(37.97–38.87)	33.87	(30.60–37.31)	0.84	(0.72–0.97)	0.81	(0.69–0.94)
South	25.30	(24.95–25.65)	27.08	(24.63–29.67)	0.82	(0.69–0.98)	0.83	(0.70–0.98)
*Urbanicity*								
Rural	67.27	(66.77–67.77)	64.47	(61.14–67.68)	1.00		1.00	
Urban	32.73	(32.23–33.23)	35.53	(32.32–38.86)	1.13	(0.99–1.30)	1.09	(0.96–1.25)

Adjusted odds ratios adjusted for age, race/ethnicity, household income, education level, region of the United States, and urbanicity. Odds ratios and confidence intervals in bold are statistically significant.

**Table 2:** Interpersonal violence among individuals who often experienced hunger during childhood in the United States.

	Often Experienced Frequent Hunger? *(“How Often Did a Parent or Other Adult Living in Your Home Make You Go Hungry or Not Prepare You Regular Meals?”)*							
	No *(Never/Almost Never/Sometimes)*		Yes *(Fairly Often/Often)*		Unadjusted		Adjusted	
	(*n* = 33,991, 98.84%)		(*n* = 436, 1.16%)					
	%	95% CI	%	95% CI	OR	95% CI	AOR	95% CI
**Violent Antisocial Behavior**								
Any violence								
No	85.41	(85.12–85.69)	63.02	(60.44–65.54)	1.00		1.00	
Yes	14.59	(14.31–14.88)	36.98	34.46–39.56)	**3.62**	**(3.20–4.10)**	**2.08**	**(1.81–2.38)**
Get into a lot of fights that you started?								
No	97.11	(96.99–97.22)	85.82	(83.31–88.01)	1.00			
Yes	2.89	(2.78–3.01)	14.14	(11.99–16.69)	**5.55**	**(4.56–6.75)**	**2.18**	**(1.77–2.68)**
Hit someone so hard that you injure them or they had to see a doctor?								
No	93.79	(93.60–93.98)	80.16	(77.67–82.44)	1.00			
Yes	6.21	(6.02–6.40)	19.84	(17.56–22.33)	**3.74**	**(3.19–4.38)**	**2.21**	**(1.85–2.64)**
Use a weapon like a stick, knife, or gun in a fight?								
No	97.27	(97.17–97.37)	86.85	(84.57–88.84)	1.00			
Yes	2.73	(2.63–2.83)	13.15	(11.16–15.43)	**5.40**	**(4.48–6.50)**	**2.34**	**(1.90–2.88)**
Physically hurt another person in any way on purpose?								
No	94.69	(94.51–94.87)	82.94	(80.90–84.80)	1.00			
Yes	5.31	(5.13–5.49)	17.06	(15.20–19.10)	**3.67**	**(3.18–4.24)**	**2.06**	**(1.79–2.38)**

Odds ratios adjusted for age, gender, race/ethnicity, household income, education level, region of the United States, urbanicity, antisocial parental history, parental drug abuse history, and clinical, personality disorders, and alcohol and drug use disorders. Odds ratios and confidence intervals in bold are significant at *p* < 0.05 or lower.

**Table 3:** Interpersonal violence among individuals who often experienced hunger during childhood, by gender.

	Female		Male	
	AOR	95% CI	AOR	95% CI
**Violent Antisocial Behavior**				
Any violence?	**1.63**	**(1.36–1.96)**	**2.73**	**(2.15–3.45)**
Get into a lot of fights that you started?	**1.41**	**(1.07–1.85)**	**3.07**	**(2.16–4.34)**
Hit someone so hard that you injure them or they had to see a doctor?	**1.92**	**(1.51–2.43)**	**2.63**	**(2.01–3.44)**
Use a weapon like a stick, knife, or gun in a fight?	**1.85**	**(1.44–2.37)**	**2.88**	**(2.01–4.13)**
Physically hurt another person in any way on purpose?	**2.09**	**(1.72–2.54)**	**2.09**	**(1.60–2.73)**

Odds ratios adjusted for age, race/ethnicity, household income, education level, region of the United States, urbanicity, antisocial parental history, parental drug abuse history, and clinical, personality disorders, and alcohol and drug use disorders. Odds ratios and confidence intervals in bold are significant at *p* < 0.05 or lower.

**Table 4:** Impulse-control problems among individuals who often experienced hunger during childhood in the United States.

	Often Experienced Frequent Hunger? *(“How Often did a Parent or other Adult Living in Your Home Make You Go Hungry or not Prepare You Regular Meals?”)*							
	No *(Never/Almost Never/Sometimes)*		Yes *(Fairly Often/Often)*		Unadjusted		Adjusted	
	(*n* = 33,991, 98.84%)		(*n* = 436, 1.16%)					
	%	95% CI	%	95% CI	OR	95% CI	AOR	95% CI
Impulse-Control Deficits								
Any self-control deficit								
No	74.24	(73.93–74.55)	48.82	(45.60–52.05)	1.00		1.00	
Yes	25.76	(25.45–26.07)	51.18	(47.95–54.40)	**3.02**	**(2.65–3.45)**	**1.97**	**(1.70–2.29)**
Have you often done things impulsively?								
No	83.12	(82.84–83.40)	65.21	(61.89–68.38)	1.00		1.00	
Yes	16.88	(16.60–17.16)	34.79	(31.62–38.11)	**2.63**	**(2.27–3.04)**	**1.70**	**(1.45–2.00)**
Have even little things made you angry or have you had difficulty controlling your anger?								
No	92.69	(92.53–92.84)	83.77	(82.15–85.27)	1.00		1.00	
Yes	7.31	(7.16–7.47)	16.23	(14.73–17.85)	**2.45**	**(2.17–2.77)**	**1.19**	**(1.04–1.36)**
Have you often had temper outbursts or gotten so angry that you lose control?								
No	93.23	(93.06–93.39)	78.57	(76.91–80.14)	1.00		1.00	
Yes	6.77	(6.61–6.94)	21.43	(19.86–23.09)	**3.75**	**(3.40–4.14)**	**1.81**	**(1.61–2.03)**
Have you hit people or thrown things when you got angry?								
No	91.28	(91.11–91.45)	76.96	(74.23–79.49)	1.00		1.00	
Yes	8.72	(8.55–8.89)	23.01	(20.51–25.77)	**3.13**	**(2.70–3.64)**	**1.66**	**(1.39–1.98)**

Odds ratios adjusted for age, gender, race/ethnicity, household income, education level, region of the United States, urbanicity, antisocial parental history, parental drug abuse history, and clinical, personality disorders, and alcohol and drug use disorders. Odds ratios and confidence intervals in bold are significant at *p* < 0.05 or lower.

## Appendix

As one might expect, the distribution of responses for the food deprivation variable is strongly skewed with the overwhelming majority of respondents reporting having “never” (93.7%) or “almost never” (3.47%) had the experience of being made to go hungry by a caretaker / not being prepared regular meals. This alone seems to suggest that the coding of this independent variable should be categorical. We did, however, spend substantial time debating whether or not those who “sometimes” (1.75%) experienced food deprivation should be coded as 0 (*i.e.*, with the “never” and “almost never” respondents) or if they should be included with those reporting they “fairly often” (0.64%) or “very often” (0.52%) experienced food neglect. In the end, we determined that conceptually it made more sense to focus on those who experienced frequent food neglect (*i.e.*, fairly often + very often) rather than on distinguishing between those who experienced very little (*i.e.*, never + almost never) and those who experienced some or more than some food neglect (*i.e.*, sometimes + fairly often + very often). We ran additional analyses in which we examined the link between food neglect and the outcome variables of interest. Specifically, we created variables in which those who reported “sometimes” experiencing hunger were contrasted with those reporting having “never” or “almost never” experienced food neglect (those who reported fairly or very often having experienced food neglect were excluded from the analyses). Using this coding approach, we found that—controlling for the sociodemographic, family history, and mental/substance use disorders—those who reported having “sometimes” experienced food neglect were only slightly more likely to have enacted one or more types of violence (AOR = 1.20, 95% CI = 1.07–1.36) and impulse control deficits (AOR = 1.12, 95% CI = 1.01–1.24). We used the same approach in coding those who reported “fairly often” experiencing food neglect *versus* those reporting “never”, “almost never”, or “sometimes” experiencing food neglect. We found that fairly often experiencing food neglect was associated with increased odds of violence (AOR = 2.03, 95% CI = 1.64–2.51) and impulse-control deficits (AOR = 1.57, 95% CI = 1.25–1.96). Finally, we did the same for those reporting that they “very often” experienced food neglect and found significant associations with violence (AOR = 2.09, 95% CI = 1.70–2.60) and impulse-control deficits (AOR = 2.59, 95% CI = 2.21–3.03). Given the pronounced differences (please note the non-overlapping confidence intervals in these contrasting coding schemes) we are confident in our approach as these analyses provide support for our coding strategy (*i.e.*, we found significant but substantively trivial findings for “sometimes” experiencing food neglect).
